# Microarray Analysis of the Gene Expression Profile and Lipid Metabolism in *Fat-1* Transgenic Cattle

**DOI:** 10.1371/journal.pone.0138874

**Published:** 2015-10-01

**Authors:** Xinfeng Liu, Chunling Bai, Xiangbin Ding, Zhuying Wei, Hong Guo, Guangpeng Li

**Affiliations:** 1 The Key Laboratory of Mammalian Reproductive Biology and Biotechnology of the Ministry of Education, Inner Mongolia University, Hohhot, China; 2 College of Animal Science and Animal Medicine, Tianjin Agriculture University, Tianjin, China; University of Lleida, SPAIN

## Abstract

Long-chain n-3 polyunsaturated fatty acids (n-3 PUFAs) are beneficial for human health. However, humans and mammals are unable to synthesize n-3 PUFAs because they lack the n-3 desaturase gene *fat-1* and must therefore obtain this type of fatty acid through their diet. Through the production of *fat-1* transgenic animals, it is possible to obtain animal products that are rich in n-3 PUFAs, such as meat and milk. The aim of this study was to analyze the gene expression profile and the mechanism of lipid metabolism in *fat-1* transgenic cattle and to accumulate important basic data that are required to obtain more efficient *fat-1* transgenic cattle. Transcriptome profiling of *fat-1* transgenic and wild-type cattle identified differentially expressed genes that are involved in 90 biological pathways, eight pathways of which were related to lipid metabolism processes 36 genes of which were related to lipid metabolism. This analysis also identified 11 significantly enriched genes that were involved in the peroxisome proliferator-activated receptor signaling pathway. These findings were verified by quantitative polymerase chain reaction. The information obtained in this study indicated that the introduction of an exogenous *fat-1* gene into cattle affects the gene expression profile and the process of lipid metabolism in these animals. These results may provide important insights into how an exogenous *fat-1* gene synthesizes n-3 PUFAs in transgenic cattle and other mammals.

## Introduction

N-3 polyunsaturated fatty acids (n-3 PUFAs) have been associated with reducing the risk of major diseases, such as cardiovascular diseases, rheumatoid arthritis, diabetes, cancer and so on [1–4]. However, due to the lack of necessary desaturases, n-3 PUFA biosynthetic pathways do not exist in humans and mammals, so n-3 PUFAs must be obtained from the diet [5,6]. The *fat-1* gene encoding the n-3 fatty acid desaturase in *Caenorhabditis elegans* has been cloned and subsequently transfected into mammalian cells, resulting in an increased cellular n-3 PUFA content [7].

Various *fat-1* transgenic animals have been generated to analyze the function of this gene. For example, Kang et al. [8] created the first *fat-1* transgenic mouse model, which was capable of producing n-3 PUFA from n-6 PUFA through the constitutive expression of the *fat-1* gene *in vivo*, reducing the ratio of n-6/n-3 fatty acids in different tissues and organs. The *fat-1* transgenic mouse model also showed that the n-3 PUFAs exert important protective effects in a variety of processes, such as bone development, inflammatory/immune pathology, cancer chemoprevention, and neurological disease [9–12]. It is generally known that the nutritional value of animal meat and milk could be increased by elevating the concentrations of n-3 PUFAs. Therefore, researchers developed other *fat-1* transgenic domestic animals. These experiments also showed that it is feasible to yield higher levels of n-3 PUFAs in transgenic animals for the purpose of improving the fatty acid composition of food products [5,13,14].

Although most of the previous studies focused on the role of the *fat-1* gene by using various transgenic animals, little is known about the mechanism by which the *fat-1* gene promotes the production of n-3 PUFAs in transgenic animals, especially transgenic cattle. Additionally, there has been little reported on the changes in gene expression patterns in *fat-1* transgenic cattle. Here, considering that blood is an important metabolic tissue that reflects the health condition of the body, we utilized cDNA microarrays to analyze the blood gene expression profiles in *fat-1* transgenic cattle. The objective of this study was to identify the significantly enriched biologically relevant pathways and to gain further insight into these biological pathways in relation to lipid metabolism in *fat-1* transgenic cattle. These results will help us accumulate important basic data that are required to obtain more efficient *fat-1* transgenic cattle.

## Materials and Methods

### Ethics statement

All studies adhered to procedures consistent with the National Research Council Guide for the Care and Use of Laboratory Animals and were approved by the Institutional Animal Care and Use Committee at Inner Mongolia University.

### Preparation of the experimental animals

All *fat-1* transgenic cattle were obtained by somatic cell nuclear transfer at the key laboratory of mammalian reproductive biology and biotechnology of the ministry of education, Inner Mongolia University, China. A total of 15 cattle (3 transgenic adult cows, 2 transgenic young bulls and 10 wild-type adult cows) were used in this study. Among these 15 cattle, 3 transgenic adult cows were produced by using the same cell line (Holstein cow fetal fibroblasts) and had a similar age (ZK002, 3.5 years; ZK005, 3.0 years; ZK006, 3.0 years) and 2 transgenic young bulls (11 months old) were produced by using another cell line of Holstein bull fetal fibroblasts; the remaining 10 wild-type Holstein cows were between 3.0 and 4.0 years old. All of the cattle were housed in a concrete-sanded cowshed for one month prior to sample collection, fed the same diet (commercial concentrate feed and wet corn silage), and monitored every day to ensure their health. In this study, we first selected 5 transgenic and 5 wild-type cattle for the analysis of blood lipids as a preliminary evaluation of the function of the *fat-1* gene, and then, 3 transgenic and 3 wild type cattle were selected for microarray analysis. Lastly, 5 transgenic and 10 wild-type cattle were selected for reverse transcription quantitative polymerase chain reaction (RT-qPCR) analysis to validate the microarray results.

### Detection of blood lipids

Blood was collected from the jugular vein, and each blood sample was placed into a tube containing 5% ethylene-diaminetetraacetic acid. Plasma was separated by centrifugation at 3500 g/min for 15 min at 4°C. Then, the concentrations of the plasma lipids, including total cholesterol (TC), triglyceride (TG), high-density lipoprotein cholesterol (HDL-C), and low-density lipoprotein cholesterol (LDL-C), were measured using a fully automatic biochemical analyzer Glamour 3000 (Misiones Bernal, Buenos Aires, Argentina).

### RNA extraction and purification

RNA was extracted from whole blood using the Trizol extraction protocol and purified using an RNeasy® Mini Kit (QIAGEN, Germany), following the manufacturer’s protocol. For quality control, total RNA was quantified using the NanoDrop ND-2000 spectrophotometer (Thermo Scientific, USA), and the RNA integrity was assessed using an Agilent Bioanalyzer 2100 (Agilent Technologies).

### Microarray hybridization

Gene expression profiling was performed using an Agilent Bovine (V2) Gene Expression microarray (4×44K) containing 43,803 probe sets that interrogate approximately 43,711 bovine transcripts from the RefSeq, Unigene, TIGR, and Btau 4.0 databases. Total RNA from each cattle was hybridized to a separate array, as previously described [15]. Briefly, total RNA was reverse-transcribed using a T7 promoter primer, and double-stranded cDNA was generated. Second-strand cDNA was synthesized, *in vitro* transcribed into cRNA, and labeled with aaUTP. Then, the cRNA was purified using a RNeasy® Mini Kit, labeled with cyanine-3-CTP, purified, and hybridized to the Gene Expression microarray. After hybridization, the microarray slides were washed once with 2×SSC, 0.1% sodium dodecyl sulfate (SDS) at 42°C for 4 min, once with 0.1×SSC, 0.1% SDS at room temperature for 10 min and three times with 0.1×SSC at room temperature for 1 min. The microarray slides were then washed with distilled water and spin-dried. The arrays were then scanned at 5 μm using the Agilent Scanner G2505C (Agilent Technologies).

### Microarray data analysis

Feature Extraction software (version10.7.1.1, Agilent Technologies) was used to analyze the array images to obtain raw data, and GeneSpring software was employed to perform the basic analysis. The raw data were normalized using the quantile algorithm. Probes specifying that at least 75% of the samples in any one out of two conditions possessed flags were employed for the selection of the genes to be used in further analyses. The gene expression data were deposited into the NCBI Gene Expression Omnibus website (http://www.ncbi.nlm.nih.gov/geo) and can be accessed via the accession number GSE66651. The differentially expressed genes were then identified through fold changes, P values were calculated using the t-test, and the false discovery rate (FDR) was calculated to correct the P values by using the R package. The threshold for up- and down-regulated genes was set as a fold change ≥1.5 and P values and FDR values <0.05. The GO and KEGG analyses were applied to determine the roles of these differentially expressed mRNAs, and the biological functions with a P value <0.05 were considered significant. Hierarchical clustering was performed to display the gene expression patterns among the samples.

### RT-qPCR verification

Reverse transcription-qPCR (RT-qPCR) was performed to validate the differentially expressed genes identified by the microarray analysis. Sixteen lipid metabolism-related genes were selected from the gene sets derived from the GO analysis and the PPAR pathway for the RT-qPCR assays. Total RNA from 5 transgenic and 10 wild-type cattle were homogenized to the same concentration. One microgram of homogenized total RNA was reverse-transcribed using random primers and oligonucleotides (dT)_18_ (GeneCopoeia Inc., USA) for cDNA synthesis, according to the manufacturer’s instructions. Then, qPCR was performed using a SYBRGreen-PCR Master kit (GeneCopoeia Inc.) in a Bio-Rad CFX Connect™ Real-Time PCR Detection System at a final volume of 20 μl. The cycling conditions were 95°C for 10 min, then 40 cycles of the following: 95°C for 10 s, 60°C for 20 s, and 72°C for 15 s. The reactions were carried out in triplicate, and the relative gene expression was expressed as a fold change and calculated by using 2^–(ΔΔCq)^. In this study, two reference genes (*GAPDH* and *β-actin*) were selected for the analysis of qPCR, and the calculation of the 2^–(ΔΔCq)^ values was performed according to the previous report of Nuruddin et al. [16]. The concrete methods were: The ΔCq was calculated from the difference in expression between the 16 target genes and the mean expression of the two reference genes (*GAPDH* and *β-actin*). The ΔΔCq was calculated by the difference between the ΔCq value of the *fat-1* transgenic cattle and the wild-type cattle samples. The mean values of three 2^–(ΔΔCq)^ were calculated as the final relative gene expression fold change. The details of the 18 genes (16 target genes and 2 reference genes), including the gene symbol, accession number, primer sequences, and product size, are listed in S1 Table.

## Results and Discussion

### Analysis of blood lipids

The blood lipid levels of the *fat-1* transgenic and wild-type cattle are shown in Table 1. The results indicated that TG, TC, HDL-C, and the ratio of TC/HDL-C were significantly decreased (p<0.05) in the *fat-1* transgenic cattle, but there was no significant influence on LDL-C and the ratio of TG/HDL-C. Several studies have shown that dyslipidemia confers a risk of coronary artery disease (CAD), while higher intakes of n-3 PUFAs are associated with a reduced risk of CAD [17]. In our study, the findings involved into the change of the plasma TG and TC levels are in line with previous studies [18]. However, we observed that the HDL-C levels were lower in *fat-1* transgenic cattle than in wild-type cattle; these results seemed to be contradictory with previous studies [19]. Kinosian et al. [20] reported that the TC/HDL-C ratio was a superior measure of risk for coronary heart disease compared with either TC or LDL-C. Therefore, we further observed the ratio of TC/HDL-C, which was significantly decreased in the *fat-1* transgenic cattle. This probably explains that the decrease of HDL-C levels is due to the significantly low TC levels in the *fat-1* transgenic cattle. In addition, it was also shown that the TG/HDL-C ratio was a significant predictor of cardiovascular disease [21], but we observed the same ratio of TG/HDL-C in the *fat-1* transgenic and wild-type cattle. A possible explanation of this finding was that the *fat-1* transgenic and wild-type cattle were healthy. However, we obtained preliminary evidence that the *fat-1* gene reduced the blood lipid levels of TG, TC, and the ratio of TC/HDL-C in transgenic cattle. These results suggested that the *fat-1* transgenic cattle might be healthier than the wild-type cattle.

**Table 1:** A comparison of the biochemical indexes of the blood lipids between *fat-1* transgenic and wild-type cattle.

Item	TG(umol/L)	TC(umol/L)	HDL-C(umol/L)	LDL-C(umol/L)	TG/HDL-C	TC/HDL-C
**Transgenic cattle**	0.14±0.06*	2.18±0.20*	1.93±0.16*	0.21±0.04	0.07±0.03	1.13±0.08*
**Wild type cattle**	0.20±0.07	3.60±1.22	2.94±0.87	0.28±0.07	0.07±0.03	1.21±0.08

Serum content of TG, TC, HDL-C, LDL-C in wide type cattle and transgenic cattle (n = 5).

*, P<0.05, transgenic cattle compared with wild-type cattle.

### Global expression profile analysis

**Fig 1:**
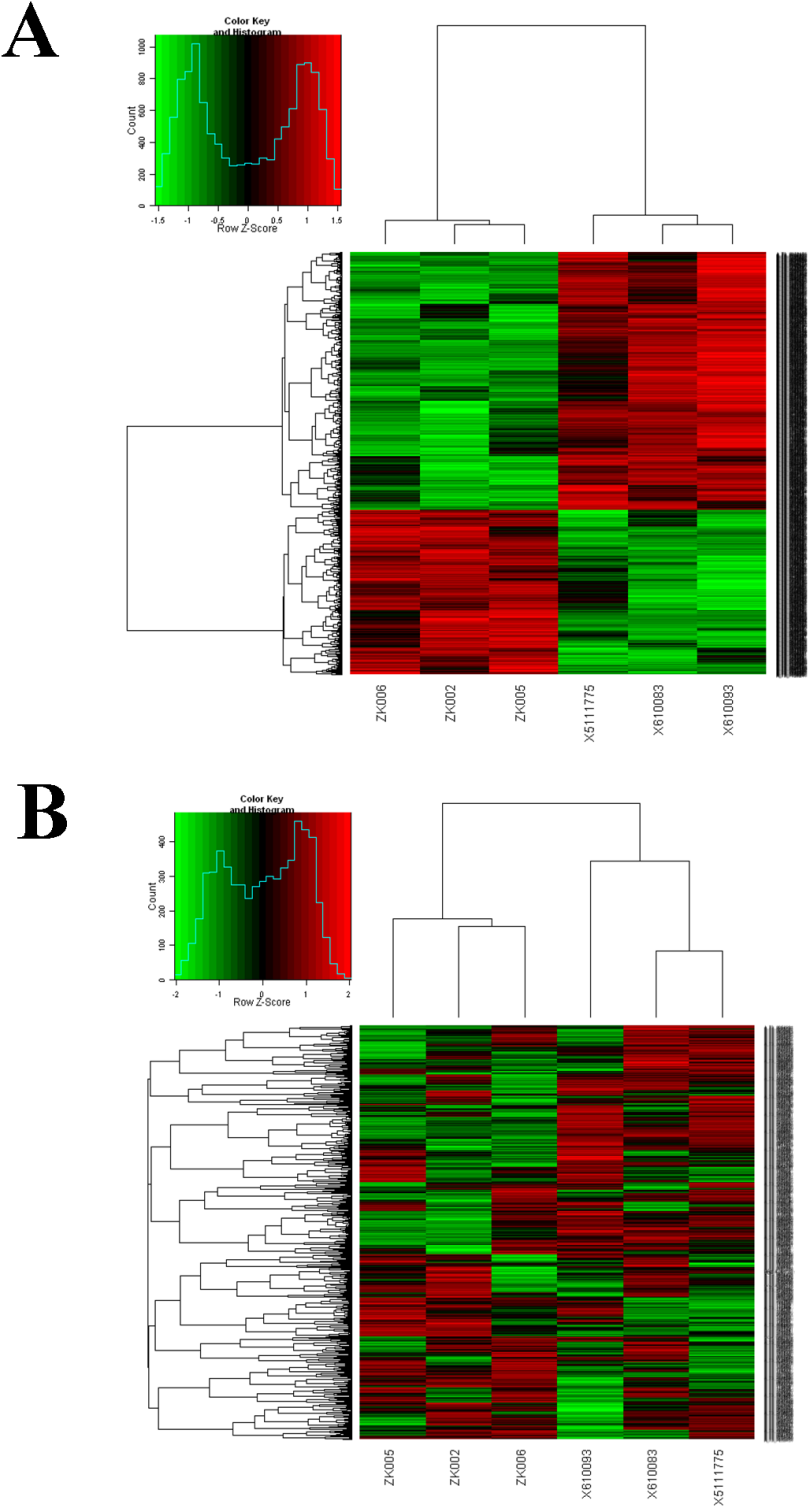
The hierarchical clustering analysis of *fat-1* transgenic cattle and wild-type cattle. (A) The heat-map of the 2042 differentially expressed genes. (B) The heat-map of the top 1000 expressed genes. The columns and rows in the heat maps represent samples and genes, respectively. Sample names are displayed below the heat maps.

In this study, we compared the gene expression patterns of the *fat-1* transgenic cattle with wild-type cattle. We used 43,711 transcript sequences as probes, and of these, 2042 were identified as being significantly differentially expressed (p<0.05, FDR<0.05), with a greater than 1.5-fold change in expression between the *fat-1* transgenic cattle and wild-type cattle. Of the 2042 transcripts, there were 797 transcripts that were significantly up-regulated in the *fat-1* transgenic cattle than in the wild-type cattle (Fig 1A, S2 and S3 Tables). Furthermore, we selected the top 1000 expressed genes for hierarchical clustering analysis and found that the transgenic cattle separated from the wild-type cattle, indicating the consistency of the genetic backgrounds for the three transgenic cattle (Fig 1B).

### Gene Ontology (GO) functional enrichment analysis of the differentially expressed genes

**Fig 2:**
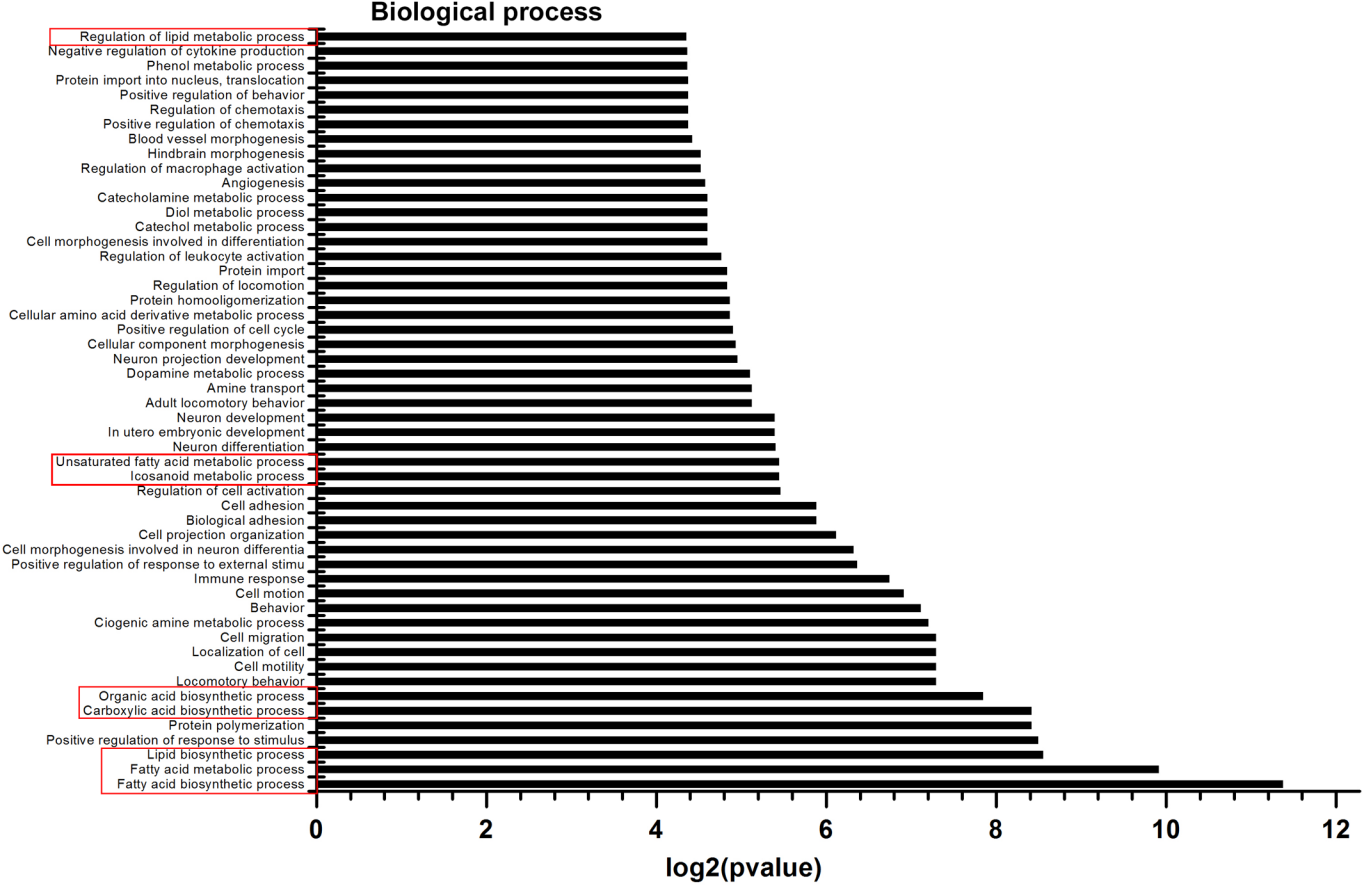
Microarray biological process (GO Ontology) classification. The x-axis indicates the likelihood [−log2(pvalue)] in a category, and the y-axis means the different subcategories of biological process. The GO terms related to lipid metabolism are represented by red boxes.

It has been suggested that PUFAs can regulate the expression of genes involved in several metabolic pathways [22]. In the present study, to gain further insight into the metabolic processes that differed between the *fat-1* transgenic and wild-type cattle, GO enrichment analysis was performed using 1605 differentially expressed genes from the 2042 significantly differentially expressed transcripts. The three GO categories (biological process, metabolic function, and cell component) were explored using DAVID bioinformatic tools for the overrepresentation of specific GO terms. To extract the most information from our gene expression data, biological functions with a P value <0.05 were considered significant. In total, 1556 differentially expressed genes were annotated in 90 GO functional groups, including 52 groups in biological processes, 15 in cellular components, and 23 in molecular functions (Fig 2, S1 and S2 Figs). In the biological process category, the most important enriched terms were related to lipid metabolism, cell behavior, and immune and nervous systems development (Fig 2). Within the cellular component category, the GO term with the highest level of significance was extracellular, including the extracellular region, extracellular region, extracellular space, and extracellular matrix (S1 Fig). Finally, calcium ion binding, phospholipase D activity, and glycosaminoglycan binding accounted for most of the terms in the molecular function category (S2 Fig). Based on the results of the GO analysis, we focused on eight GO terms for the biological process related to lipid metabolism (Fig 2). Of these, ‘Fatty acid metabolic process’ and ‘Fatty acid biosynthetic process’ are mainly involved in liberating fatty acids from naturally occurring fats and oils by hydrolysis. ‘Lipid biosynthetic process’, ‘Carboxylic acid biosynthetic process’, and ‘Organic acid biosynthetic process’ exert important role in the formation of lipids, carboxylic acids, and organic acids, respectively. ‘Icosanoid metabolic process’ and ‘Unsaturated fatty acid metabolic process’ are the main metabolic processes representing the chemical reactions and pathways involving an unsaturated fatty acid. ‘Regulation of lipid metabolic process’ can modulate the frequency, rate or extent of the chemical reactions and pathways involving lipids. This indicated that these eight lipid metabolic processes may be involved in the function of the *fat-1* gene.

### Genes related to lipid metabolism

To gain further insight into the genes related to lipid metabolism in the *fat-1* transgenic cattle, we extracted these genes from the eight GO terms of the lipid metabolism processes obtained by the GO enrichment analysis (S4 Table). We found 36 significantly differentially expressed genes involved in eight lipid metabolism processes; 17 of these genes were up-regulated in the *fat-1* transgenic cattle (Table 2) and 19 of these genes were down-regulated (Table 3). Of these 36 differentially expressed genes, eight (*CYP51A1*, *MSMO1*, *HMGCS1*, *HMGCR*, *FDFT1*, *CYP39A1*, *CH25H*, and *APOA1*) are involved in cholesterol biosynthetic process. Eight genes (*SCD5*, *LPL*, *MSMO1*, *LOC615051*, *PLP1*, *AGMO*, *FASN*, and *CH25H*) are involved in fatty acid biosynthetic process. Four genes (*MSMO1*, *CIDEA*, *SNCA*, and *FABP3*) are involved in fatty acid metabolic process. Three genes (*ACOX1*, *PEX5*, and *CPT1B*) are involved in fatty acid oxidation. Four genes (*CIDEA*, *AGMO*, *CH25H*, and *PTDSS2*) are involved in lipid metabolic process. Finally, the remaining genes are involved in the other biological processes related to lipid metabolism. The *LOC782922* is involved in the prostaglandin biosynthetic process. The *AGPAT41* is involved in the CDP-diacylglycerol biosynthetic process. The *STAT5A* is involved in lipid storage. This indicated that these significantly differentially expressed genes can influence the same or different biological processes related in lipid metabolism.

**Table 2:** The significantly up-regulated lipid metabolism-related genes in *fat-1* transgenic cattle.

GeneSymbol	Description	GenbankAccession	p-value	Fold Change
CYP51A1	Bos taurus cytochrome P450,family 51,subfamily A,polypeptide 1	NM_001025319	0.024	1.727
*ACOX1	Bos taurus acyl-CoA oxidase 1,palmitoyl	NM_001035289	0.019	1.589
*SCD5	Bos taurus stearoyl-CoA desaturase 5	NM_001076945	0.010	1.786
AGPAT4	Bos taurus cDNA clone IMAGE:8166104	BC114144	0.006	2.240
ALOX5AP	Bos taurus arachidonate 5-lipoxygenase-activating protein	NM_001076293	0.033	1.584
*LPL	Bos taurus lipoprotein lipase	NM_001075120	0.028	5.407
LOC782922	Bos taurus prostaglandin F synthetase II-like	NM_001166224	0.002	5.961
MSMO1	Bos taurus methylsterol monooxygenase 1	NM_001098863	0.019	2.094
HMGCS1	Bos taurus HMGCS1 protein-like	NM_001206578	0.007	1.843
PRODH	Bos taurus proline dehydrogenase (oxidase) 1	NM_001075185	0.041	1.583
PEX5	Peroxisomal targeting signal 1 receptor	BT029859	0.002	8.953
HMGCR	Bos taurus 3-hydroxy-3-methylglutaryl-CoA reductase	NM_001105613	0.002	1.626
FDFT1	Bos taurus farnesyl-diphosphate farnesyltransferase 1	NM_001013004	0.001	1.692
GGPS1	Bos taurus geranylgeranyl diphosphate synthase 1	NM_001079801	0.010	1.805
CIDEA	Bos taurus cell death-inducing DFFA-like effector	NM_001083449	0.0164	1.549
IFNG	Bos taurus interferon, gamma	NM_174086	0.0234	6.972
NR3C1	Bos taurus nuclear receptor subfamily 3, group C, member 1 (glucocorticoid receptor)	NM_001206634	0.0352	2.106

The genes marked with an asterisk are the same as the 11 genes from the PPAR signaling pathways by the KEGG enrichment analysis.

**Table 3:** The significantly down-regulated lipid metabolism-related genes in *fat-1* transgenic cattle.

GeneSymbol	Description	GenbankAccession	p-value	Fold Change
LOC615051	Uncharacterized protein	XM_002693733	0.040	1.768
*CPT1B	Bos taurus carnitine palmitoyltransferase 1B (muscle)	NM_001034349	0.024	1.950
CYP39A1	Bos taurus cytochrome P450, family 39, subfamily A, polypeptide 1	NM_001098938	0.012	1.824
STAT5A	Bos taurus signal transducer and activator of transcription 5A	NM_001012673	0.011	1.588
MIF	Bos taurus macrophage migration inhibitory factor (glycosylation-inhibiting factor)	NM_001033608	0.044	1.888
PLP1	Bos taurus proteolipid protein 1	NM_174149	0.012	2.035
ALOX12B	Bos taurus arachidonate 12-lipoxygenase, 12R type	NM_001192038	0.005	2.651
RNPEP	Bos taurus arginyl aminopeptidase (aminopeptidase B)	NM_001097563	0.028	1.771
AGMO	Bos taurus alkylglycerol monooxygenase	NM_001192973	0.030	2.331
PYCR1	Bos taurus pyrroline-5-carboxylate reductase 1	NM_001014957	0.044	1.593
FASN	Bos taurus fatty acid synthase	NM_001012669	0.010	1.778
EDN1	Bos taurus endothelin 1	NM_181010	0.036	4.768
ISYNA1	Bos taurus inositol-3-phosphate synthase 1	NM_001046032	0.045	1.883
CH25H	Bos taurus cholesterol 25-hydroxylase	NM_001075243	0.022	2.790
SNCA	Bos taurus synuclein, alpha (non A4 component of amyloid precursor)	NM_001034041	0.000	2.491
*APOA1	Bos taurus apolipoprotein A-I	NM_174242	0.011	2.196
PTDSS2	PREDICTED: Bos taurus phosphatidylserine synthase 2	XM_608287	0.002	4.543
*FABP3	Bos taurus fatty acid binding protein 3, muscle and heart (mammary-derived growth inhibitor)	NM_174313	0.041	1.747
PDGFA	Bos taurus platelet-derived growth factor alpha polypeptide	NM_001075231	0.036	3.215

The genes marked with an asterisk are the same as the 11 genes from the PPAR signaling pathways by the KEGG enrichment analysis.

In the present study, we further focused on four key genes (*LPL*, *FASN*, *SCD5*, and *ACOX1*) related to lipolytic and lipogenic processes. We detected that the expression level of *LPL* increased by more than five times in *fat-1* transgenic cattle relative to wild-type cattle (Table 2). It has been reported that supplementation of n-3 PUFAs in the diet can increase the transcription rate of LPL and accelerate plasma TG clearance [23]. FASN is another key enzyme of lipid metabolism, which plays an important role in *de novo* lipogenesis in mammals [24]. One study reported that n-3 PUFAs can decrease *FASN* gene expression and enzyme activity in bovine muscle [25]. Our study shows that there is decreased expression of *FASN* in *fat-1* transgenic cattle compared to wild-type cattle (Table 3). Our study also showed that the other two key genes related to lipid metabolism, *SCD5* and *ACOX1*, were up-regulated in *fat-1* transgenic cattle (Table 2), which is consistent with previous reports [26,27]. The regulation of *SCD* expression by PUFAs has also been observed in brain and immune tissues [28,29]. Furthermore, the over-expression of *SCD* can change the fatty acid composition, such as increasing the proportion of C18:2 (linoleic acid), C18:3 (linolenic acid), C20:1, C20:3, C20:4 (arachidonic acid), and C20:5 (eicosapentaenoic acid) and reducing the proportion of C14:0 (myristic acid) and total saturated fatty acids in C2C12 cells [26]. Therefore, improving the proportion of PUFAs may require changes in fatty acid metabolism or uptake by the up-regulation of the expression of *SCD* [26]. ACOX1 is the first enzyme in peroxisomal fatty acid β-oxidation; it is rate-limiting and plays a key role in fatty acid metabolism and fat deposition [30]. In *ACOX1* null mice, the expression of *ACOX1* was accompanied by increased arachidonic acid (20:4) and DHA (22:6) levels in the serum [31]. Furthermore, older *fat-1* mice showed a significant decrease in body weight and epididymal fat mass and an increased expression of *ACOX1* [27]. Thus, the function of *ACOX1* is potentially related to fat deposition, given its important role in lipid metabolism [31].

Taken together, although we did not analyze and discuss in detail all of the differentially expressed genes related to lipid metabolism, our study indicated that many key genes related to lipid metabolism were influenced in *fat-1* transgenic cattle, suggesting that the *fat-1* gene could change the expression of lipid metabolism genes.

### PPAR pathway affected by the differentially expressed genes

**Fig 3:**
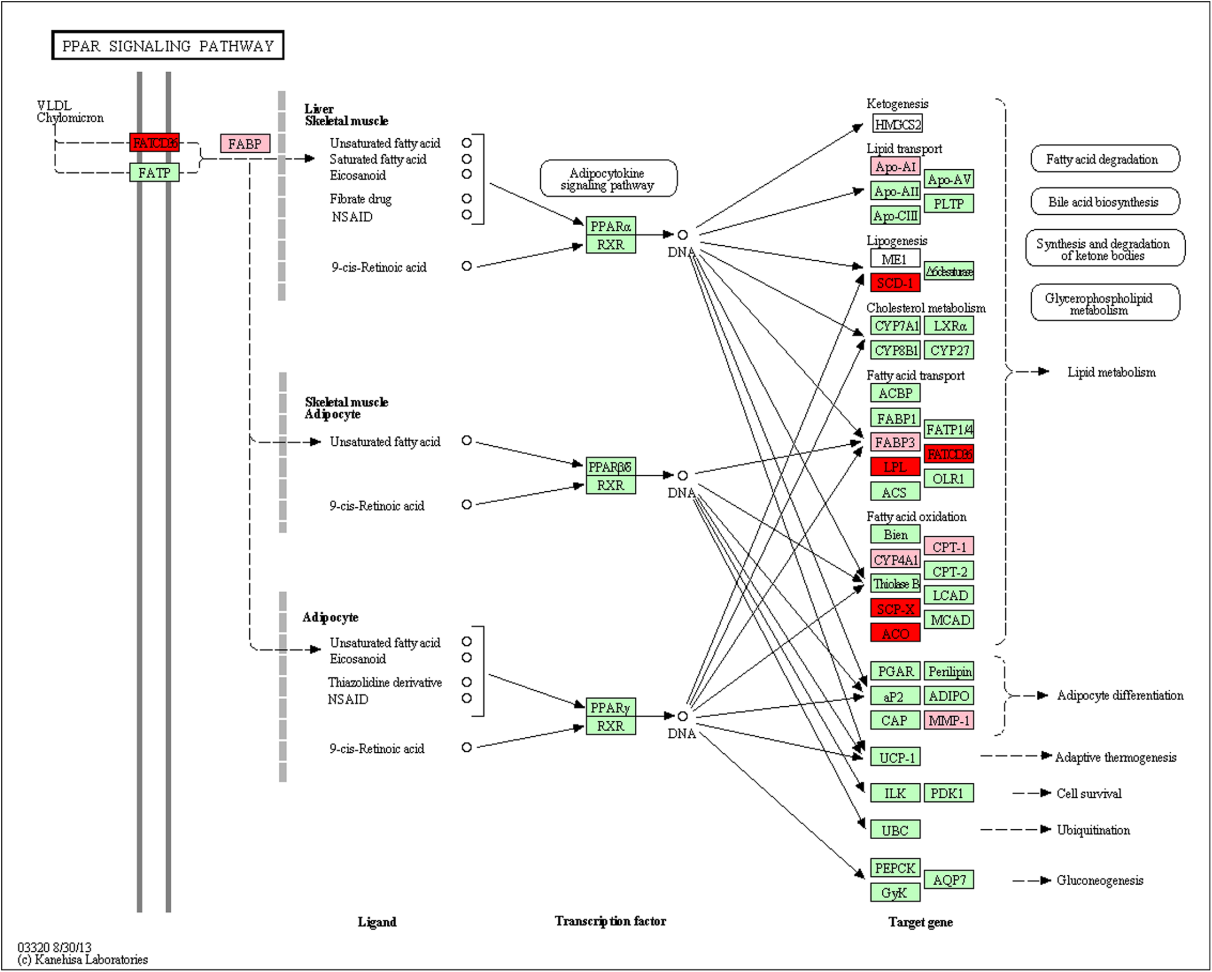
The significantly enriched genes in the ‘PPAR signaling pathway’. Red nodes indicate the significantly up-regulated genes in *fat-1* transgenic cattle, and the pink nodes indicate the significantly down-regulation genes.

Pathway-based analysis helps to further understand the biological functions of genes. In this study, we performed KEGG analysis and obtained two significantly enriched signal pathways, including the ‘ECM-receptor interaction’ and ‘PPAR signaling pathway’ (p<0.05, FDR<0.2). The ‘PPAR signaling pathway’ is important during adipocyte tissue development and differentiation and the activation of lipogenesis [32]. It is clear that long-chain PUFAs can activate PPARs and subsequently regulate the expression of important genes that are related to lipid metabolism [33]. Therefore, we focused on the ‘PPAR signaling pathway’. We observed that there were 11 significantly differentially expressed genes enriched in the ‘PPAR signaling pathway’ (Fig 3 and S5 Table). When *fat-1* transgenic cattle were compared with wild-type cattle, there was a significant (P < 0.05) increase in the expression of six genes related to lipid metabolism (*ACOX1*, *SCP2*, *FABP2*, *CD36*, *SCD5*, and *LPL*) and a significant (P < 0.05) decrease in the expression of five other genes (*MMP1*, *CPT1B*, *CYP4A22*, *FABP3*, and *APOA1*) (Fig 3 and S5 Table). At the same time, we found that 6 of the 11 genes (*FABP3*, *APOA1*, *CPT1B*, *ACOX1*, *SCD5*, *LPL*) were same as the 36 genes from the eight GO terms of the lipid metabolism processes obtained by GO enrichment analysis (Table 2, Table 3 and S5 Table).

Of the 11 differentially expressed genes enriched in the ‘PPAR signaling pathway’, four (*FABP2*, *FABP3*, *CD36* and *LPL*) are involved in fatty acid transport. Four genes (*CPT1B*, *CYP4A22*, *SCP2* and *ACOX1*) are involved in fatty acid oxidation. Finally, of the remaining three genes, *APOA1*, *SCD5* and *MMP1* are involved in lipid transport, lipogenesis, and adipocyte differentiation, respectively. Our findings showed that *fat-1* influences the ‘PPAR signaling pathway’, and the 11 differentially expressed genes point to its important role in the regulation of an extensive network of genes that are involved in lipid metabolism.

### Quantitative PCR validation of the microarray results

**Fig 4:**
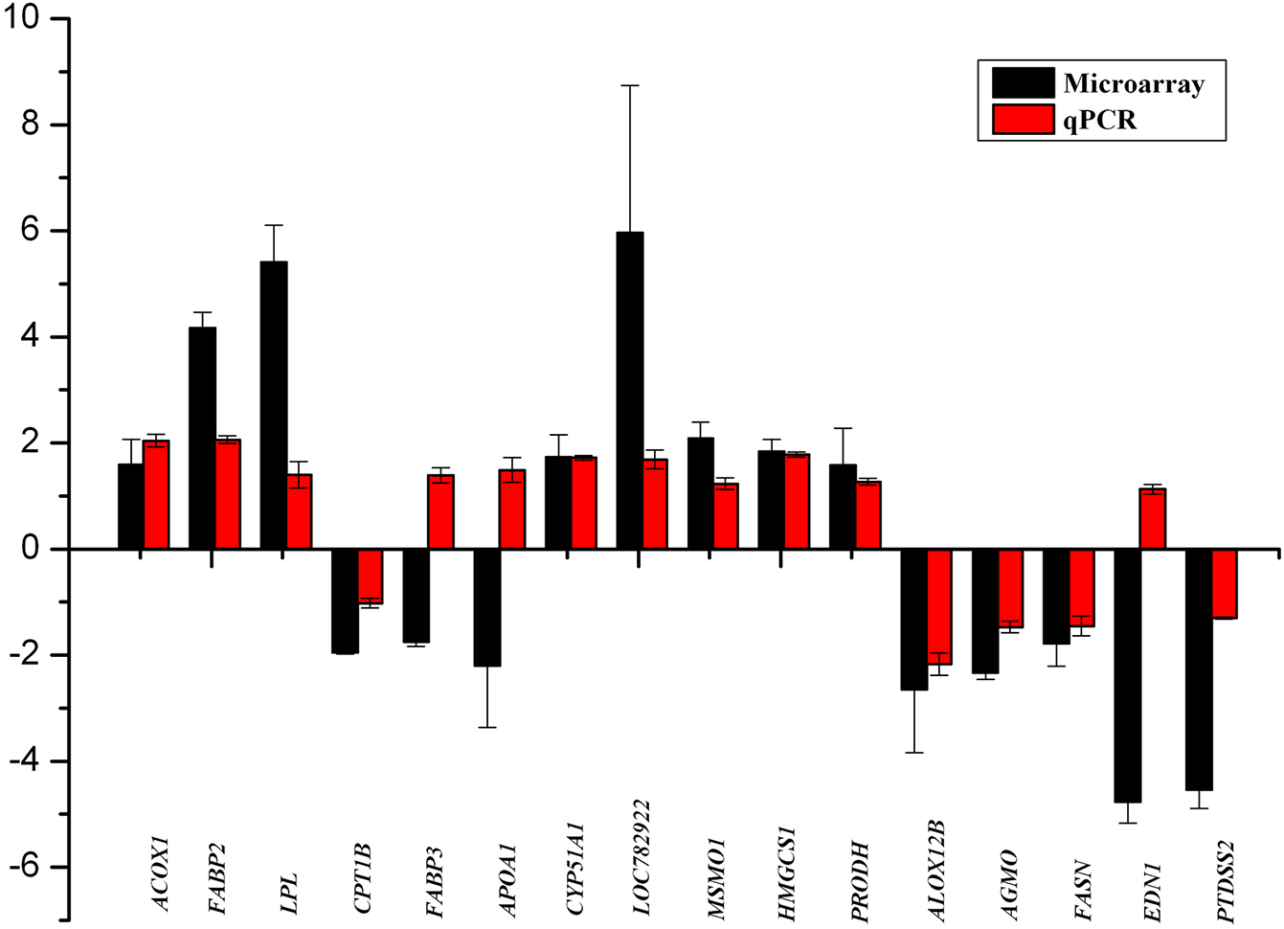
Validation of sixteen microarray differentially expressed genes by RT-qPCR. The fold-change value is expressed as positive when the genes are highly expressed in transgenic cattle and as negative when the genes are highly expressed in wild-type cattle. The gene names are displayed below the histogram.

To confirm the gene expression patterns obtained by the microarray analysis, 16 genes were examined by RT-qPCR. We first analyzed 6 of the 11 differentially expressed genes in the PPAR pathway (*ACOX1*, *CPT1B*, *APOA1*, *FABP3*, *FABP2*, *LPL*). The results showed that four genes (*ACOX1*, *CPT1B*, *FABP2*, *LPL*) were in agreement with the microarray data, with the exception of the *APOA1* and *FABP3* genes (Fig 4). The *APOA1* gene encodes apolipoprotein AI (apo AI), which is the major protein component of HDL and plays a key role in lipid metabolism and transport [34]. Despite the discrepancy observed in our study regarding the expression of *APOA1*, our RT-qPCR results were consistent with the results of previous studies showing that the levels of apolipoprotein increases in the body through the consumption of high n-3 PUFA-containing foods [35]. FABP3 is a small cytoplasmic protein, and it is involved in fatty acid metabolism because it can transport fatty acids into the mitochondria from the cell membrane. This protein is proposed to be involved in early myocardial development and adult myocardial tissue repair and is thought to be responsible for the modulation of cell growth and proliferation [36]. Our RT-qPCR results also demonstrated the high expression of *FABP3* in the *fat-1* transgenic cattle, suggesting that the *fat-1* gene potentially plays a role in the regulation of the expression of the *FABP3* genes. We then analyzed 10 of the 32 differentially expressed genes related to lipid metabolism as obtained by GO enrichment analysis (*CYP51A1*, *LOC782922*, *MSMO1*, *HMGCS1*, *PRODH*, *ALOX12B*, *AGMO*, *FASN*, *EDN1*, *PTDSS2*). Except for *EDN1*, both the microarray results and the RT-qPCR data showed that *CYP51A1*, *LOC782922*, *MSMO1*, *HMGCS1*, and *PRODH* were up-regulated and that *ALOX12B*, *AGMO*, *FASN*, and *PTDSS2* were down-regulated in *fat-1* transgenic cattle (Fig 4). EDN1 is a potent vasoconstrictor. The beneficial and detrimental physiological roles of EDN1 were reported in different studies. One study reported that high concentrations of EDN1 might lead to the constriction of coronary arteries, thereby impairing the contractility of the heart, resulting in a dilated cardiomyopathy (DCM) phenotype [37]. However, the present study reported that decreasing the expression of *EDN1* by as little as 35% caused severely dilated cardiomyopathy, while a threefold increase in the expression of EDN1 only caused slight cardiac hypertrophy, suggesting that cardiac function was sensitive to even modest changes in EDN1 levels [38]. Our RT-qPCR result indicated that the expression of *EDN1* was slightly elevated in *fat-1* transgenic cattle, which was inconsistent with our microarray results. However, we speculated that the expression increase of *EDN1* may be modest and beneficial for the health of the transgenic cattle, especially for maintaining the normal contractile functions of the heart and blood vessels.

Taken together, the results showed that 13 of the 16 genes analyzed showed the same expression patterns between the RT-qPCR and the microarray analysis (Fig 4), indicting a high consistency between both analyses, confirming the reliability of the microarray data.

In conclusion, we used microarray technology to analyze the differences in gene expression between *fat-1* transgenic cattle and wild-type cattle. We identified 36 differentially expressed genes belonging to eight biological pathways related to lipid metabolism processes. Furthermore, we found that exogenous *fat-1* can influence an important lipid metabolism signaling pathway, the PPAR signaling pathway, and 11 genes were significantly enriched in this signaling pathway. Therefore, our findings provide further insight into the function of the *fat-1* gene and how to endogenously synthesize n-3 PUFAs in transgenic animals.

## Supporting Information

S1 Fig Microarray cellular component (GO Ontology) classification. The x-axis indicates the likelihood [−log2(pvalue)] in a category, and the y-axis indicates the different subcategories of cellular components.
(TIF)

S2 Fig Microarray molecular function (GO Ontology) classification. The x-axis indicates the likelihood [−log2(pvalue)] in a category, and the y-axis means the different subcategories of molecular function.
(TIF)

S1 Table Primers used in the relative quantification of the selected genes from the microarray data. (DOC)

S2 Table Discrepancy of the expression and up regulation of transcripts in the *fat-1* transgenic cattle. (XLS)

S3 Table Discrepancy of the expression and down regulation of transcripts in the *fat-1* transgenic cattle. (XLS)

S4 Table The information regarding the significantly enriched genes in each GO term related to lipid metabolism. (XLS)

S5 Table The significantly enriched genes in the ‘PPAR signaling pathway’. (DOC)
