# Supplementary figures and images for: Microarray Analysis of the Gene Expression Profile and Lipid Metabolism in Fat-1 Transgenic Cattle
Source: PLoS One. 2015 Oct 1;10(10):e0138874. doi: 10.1371/journal.pone.0138874 (PMC4591129; doi:10.1371/journal.pone.0138874)

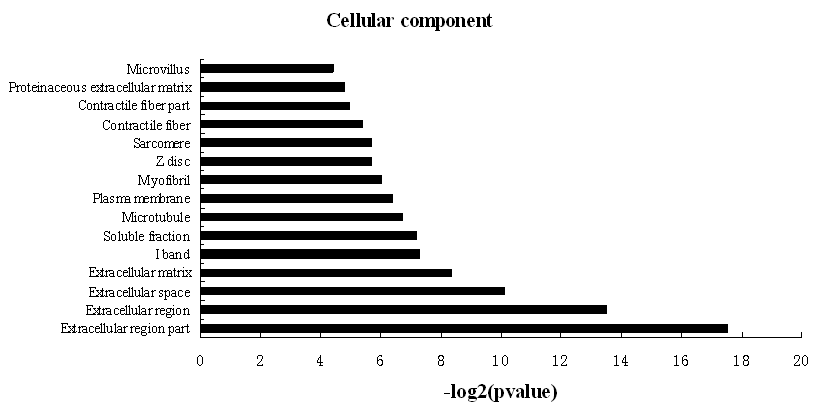

Supplement: S1 Fig — The x-axis indicates the likelihood [−log2(pvalue)] in a category, and the y-axis indicates the different subcategories of cellular components. (TIF) [file pone.0138874.s001.tif]

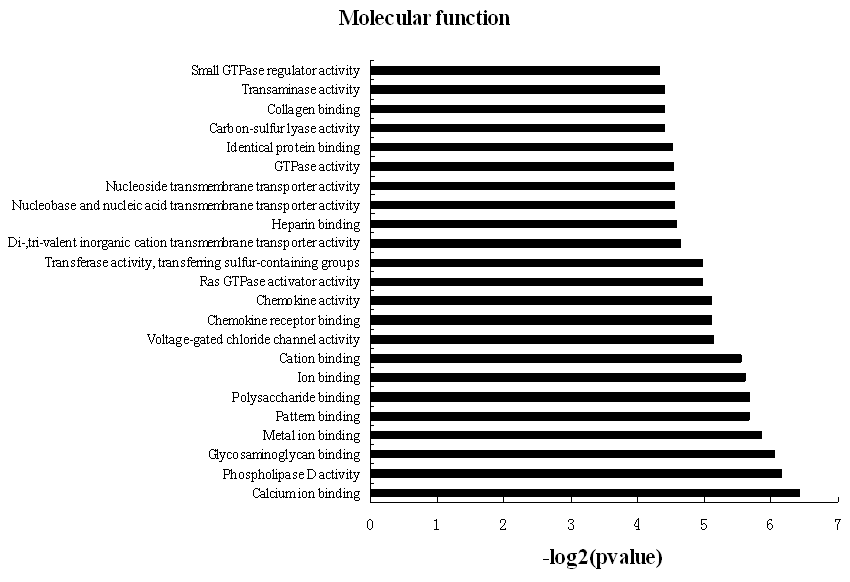

Supplement: S2 Fig — The x-axis indicates the likelihood [−log2(pvalue)] in a category, and the y-axis means the different subcategories of molecular function. (TIF) [file pone.0138874.s002.tif]
