# Supplementary material for: Microarray Analysis of the Gene Expression Profile and Lipid Metabolism in Fat-1 Transgenic Cattle
Source: PLoS One. 2015 Oct 1;10(10):e0138874. doi: 10.1371/journal.pone.0138874 (PMC4591129; doi:10.1371/journal.pone.0138874)
Supplement: S1 Table — (DOC) [file pone.0138874.s003.doc]

**S1 Table. Primers used in the relative quantification of the selected genes from the microarray data.**

| Gene symbol | Accession number | Primer sequence | Product length |
| --- | --- | --- | --- |
| *GAPDH* | NM_001034034.2 | F 5’- TGTTGTGGATCTGACCTGCC -3’ | 135 |
|  |  | R 5’- AAGTCGCAGGAGACAACCTG -3’ |  |
| *β-actin* | NM_173979.3 | F 5’- CCAACTGGGACGACATGGAG -3’ | 162 |
|  |  | R 5’- CAGGGGTGTTGAAGGTCTCG -3’ |  |
| *ACOX1* | NM_001035289.3 | F 5’- GGCGGACATGGCTATTCTCA -3’ | 91 |
|  |  | R 5’- TGACAGTGTTTTCCCCCTCG -3’ |  |
| *CPT1B* | NM_001034349.2 | F 5’- CGTTCTTCGTGGCTCTGGAT -3’ | 122 |
|  |  | R 5’- GGACTTGTCGAACCACCTGT -3’ |  |
| *FABP3* | NM_174313.2 | F 5’- GACCACAGCAGATGACAGGA -3’ | 113 |
|  |  | R 5’- CAACCATCTCCCGCACAAGT -3’ |  |
| *APOA1* | NM_174242.3 | F 5'- GAGACTGCTGGCCATTGAGGT -3' | 129 |
|  |  | R 5'- GGATGACTGGGGGTCATCTTG -3' |  |
| *FABP2* | NM_001025332.1 | F 5'- GGCGAGATGGTCCAGACTTAC -3' | 146 |
|  |  | R 5'- TGAGGAAAGGAAAGCATTTTTGGG -3' |  |
| *LPL* | NM_001075120.1 | F 5’- GGACAGGATGTGGCCAAGTT -3’ | 150 |
|  |  | R 5’- GCCGGTTATCCTGTTGACCT -3’ |  |
| *CYP51A1* | NM_001025319.2 | F 5’- TGCCGAGGAAGTCTACAGTC -3’ | 137 |
|  |  | R 5’- CGTGCTGTCTAAAGTGGGCTA -3’ |  |
| *LOC782922* | NM_001166224.1 | F 5’- AAGGGTATTGGTCACCCTGAGT -3’ | 100 |
|  |  | R 5’- TCGTAGCCCTAGAAGCAGACG -3’ |  |
| *MSMO1* | NM_001098863.1 | F 5’- CGGTCTGGCGGCAGAATAAT -3’ | 135 |
|  |  | R 5’- CTGCAGAGGATTGTCAGGCA -3’ |  |
| *HMGCS1* | NM_001206578.1 | F 5’- AGGCCTAGTGAAACTGGCAC -3’ | 140 |
|  |  | R 5’- TTCAGGATGTTAGTTCAGGAGGG -3’ |  |
| *PRODH* | NM_001075185.1 | F 5’- CCCGCACCTACTTCTATGCC -3’ | 139 |
|  |  | R 5’- AACTGTGGTCTCCCCAAAGC -3’ |  |
| *ALOX12B* | NM_001192038.1 | F 5’- ACATTTACCTGGCCGACTACC -3’ | 126 |
|  |  | R 5’- TAGGCAGGAGGTTCCCTTCC -3’ |  |
| *AGMO* | NM_001192973.1 | F 5’- CTTTCCGTTGCTTGGTGTTCC -3’ | 129 |
|  |  | R 5’- TCACACTCCTTACTCCCCAGAA -3’ |  |
| *FASN* | NM_001012669.1 | F 5’- TCAGGAGTTTCCCTCACTTACC -3’ | 140 |
|  |  | R 5’- CCAACCTCTTCCATGAGCCA -3’ |  |
| *EDN1* | NM_181010.2 | F 5’- AACAGCAATCAGGCCGAAGT -3’ | 131 |
|  |  | R 5’- TCCCCCTGCAAACTAAACTCG -3’ |  |
| *PTDSS2* | XM_608287.5 | F 5’- GCACCAGCTACCCAACTTCA -3’ | 103 |
|  |  | R 5’- CTCGAGGGTCTTCATGCCAC -3’ |  |

F indicates forward prime; R indicates reverse primer.
