# Supplementary material for: Microarray Analysis of the Gene Expression Profile and Lipid Metabolism in Fat-1 Transgenic Cattle
Source: PLoS One. 2015 Oct 1;10(10):e0138874. doi: 10.1371/journal.pone.0138874 (PMC4591129; doi:10.1371/journal.pone.0138874)
Supplement: S5 Table — (DOC) [file pone.0138874.s007.doc]

**S5 Table. The significantly enriched genes in the** ‘**PPAR signaling pathway**’**.**

| **GeneSymbol** | **Description** | **GenbankAccession** | **Regulation** | **p-value** | **Fold Change** |
| --- | --- | --- | --- | --- | --- |
| *FABP3 | Bos taurus fatty acid binding protein 3, muscle and heart (mammary-derived growth inhibitor) | NM_174313 | down | 0.041 | 1.747 |
| *APOA1 | Bos taurus apolipoprotein A-I | NM_174242 | down | 0.011 | 2.196 |
| MMP1 | Bos taurus matrix metallopeptidase 1 | NM_174112 | down | 0.013 | 2.533 |
| *CPT1B | Bos taurus carnitine palmitoyltransferase 1B (muscle) | NM_001034349 | down | 0.024 | 1.950 |
| CYP4A22 | Bos taurus cytochrome P450, family 4, subfamily A, polypeptide 22 | NM_001098990 | down | 0.027 | 1.607 |
| *ACOX1 | Bos taurus acyl-CoA oxidase 1, palmitoyl | NM_001035289 | up | 0.019 | 1.589 |
| SCP2 | Bos taurus sterol carrier protein 2 | NM_001033990 | up | 0.045 | 1.550 |
| FABP2 | Bos taurus fatty acid binding protein 2, intestinal | NM_001025332 | up | 0.043 | 4.167 |
| CD36 | Bos taurus CD36 molecule (thrombospondin receptor) | NM_174010 | up | 0.015 | 1.960 |
| *SCD5 | Bos taurus stearoyl-CoA desaturase 5 | NM_001076945 | up | 0.010 | 1.786 |
| *LPL | Bos taurus lipoprotein lipase | NM_001075120 | up | 0.028 | 5.407 |

The genes marked with an asterisk are the same as the 32 genes from the eight GO terms of lipid metabolism processes by the GO enrichment analysis.
